# Clinical outcomes in eyes with diffractive continuous depth-of-focus intraocular lenses enhanced for near vision: comparison with trifocal intraocular lenses

**DOI:** 10.1186/s12886-023-03207-6

**Published:** 2023-11-21

**Authors:** Yuya Nomura, Yuka Ota, Yoshifumi Fujita, Tomohisa Nishimura, Hiroko Bissen-Miyajima, Keiichiro Minami

**Affiliations:** 1 Fujita Eye Clinic, Tokushima, Japan; 2 Department of Ophthalmology, Tokyo Dental College Suidobashi Hospital, Tokyo, Japan; 3 Mikawa Eye Clinic, Saga, Japan

**Keywords:** Continuous depth-of-focus intraocular lens, Trifocal intraocular lens, Binocular visual acuity, Binocular contrast sensitivity, Intermediate distance, Pelli-Robson chart

## Abstract

**Background:**

To prospectively evaluate visual functions and patient satisfaction after bilateral implantation of diffractive continuous depth-of-focus intraocular lens (CDF IOL) compared with trifocal IOLs.

**Methods:**

This investigator-initiated study was approved by a certified local review board (registered: jRCTs032210305). CDF IOL (Synergy, J&J, group S) and trifocal IOL (AcrySof PanOptix, Alcon, group P) were implanted bilaterally in 30 patients each. Three months postoperatively, binocular outcomes of uncorrected (BUCVA) and distance-corrected (BDCVA) visual acuities at distances of 0.3, 0.4, 0.5, 0.7, and 5 m were measured. Contrast sensitivities were binocularly measured using CSV-1000 (2.5 m) and Pelli-Robson charts at distances of 0.4 and 1 m. Symptoms of glare, halo, starburst, and waxy vision, and satisfaction for near, intermediate, and far visions were assessed with questionnaires. Differences between the two groups were examined.

**Results:**

Twenty-seven patients each completed the follow-up. The mean age of the group S was lower than that of the group P (P < 0.001). The BUCVA at 0.4 m was better in the S group, while the mean manifest refraction of the P group showed a significant hyperopic shift (P < 0.001). BDCVA was significantly better in the S group. The contrast sensitivity results at three distances showed no discernible differences. Although more patients in the S group reported significant glare and halo, their satisfaction with near vision was higher.

**Conclusions:**

The binocular visual function of patients with CDF IOLs was comparable to or better than that of patients with trifocal IOLs. The patients were satisfied with near vision, despite the enhanced glare and halo. Understanding the differences between the two types of presbyopia-correcting IOLs is important to ensure patient satisfaction.

**Trial registration:**

This clinical trial was registered in the Japan Registry for Clinical Research (identifier: jRCTs032210305) on September 13, 2021.

## Background

Presbyopia-correcting intraocular lenses (IOLs) are used to achieve independence from or reduce the dependence on spectacles after cataract surgery. With the use of bifocal IOLs, patients obtain uncorrected vision at both far and near distances but their visual acuity is often degraded at intermediate distances. Diffractive trifocal IOLs have been developed to demonstrate acceptable visual acuity from near to far distances by adding foci at near and intermediate distances [1, 2]. A worldwide prospective investigation of 1094 eyes of 557 patients with diffractive trifocal PanOptix^®^ IOLs TFNT00 (Alcon laboratories, Fort Worth, TX) shows that visual acuities of 0.1 logarithm of the minimum angle of resolution (logMAR) or better were continuously obtained from distance to near [3]. Alternatively, continuous vision from distance to near can be achieved by combining extended depth-of-focus and bifocal profiles. In a study by Ribeiro et al. [4], binocular visual acuities of 0.1 logMAR or better were continuously obtained between far (+ 0.50 D) and near (-3.00 D) in eyes with continuous depth-of-focus (CDF) IOLs (Synergy® ZFR00V, Johnson & Johnson Vision, Santa Ana, CA). Because the two types of IOLs are based on distinct optical designs, their optical performances are inherently different [5]. Postoperative visual acuity, defocus curve, contrast sensitivity at far distances, and photic phenomena were well comparable between the two types of IOLs [6–8]. However, to our knowledge, previous studies have evaluated contrast sensitivity only at far distances, whereas it is important to investigate visual functions at intermediate and near distances to understand patient satisfaction at each vision distance. Hence, this prospective multisite open-label study aimed to evaluate visual function and patient satisfaction after bilateral implantation of CDF IOLs.

## Methods

### Participants

This investigator-initiated prospective comparative study was approved by the local Certified Review Board (Shinanozaka Clinic/Hattori Clinic CRBs, Tokyo, Japan) and registered with the Japan Registry for Clinical Research (identifier: jRCTs032210305). This study was conducted in accordance with the tenets of the Declaration of Helsinki and Clinical Trials Act of Japan (Act No. 16, 2017). Written informed consent was obtained from all patients. Patients who underwent bilateral cataract surgery with bilateral implantation of multifocal IOLs were recruited from Fujita Eye Clinic (Tokushima, Japan), Mikawa Eye Clinic (Saga, Japan), and Tokyo Dental College Suidobashi Hospital (Tokyo, Japan). The inclusion criteria were age 60–79 years and a target refraction of emmetropia. Patients with other ocular diseases influencing visual function (e.g., uveitis, acute ocular disease, external/internal infection, diabetic retinopathy, glaucoma, exfoliation syndrome, pathological miosis, keratoconus, corneal endothelial dystrophy, and weak zonules), a history of intraocular or corneal surgery, or other systemic or ophthalmic diseases unsuitable for this study were excluded.

### Sample size

The sample size was determined to evaluate binocular distance-corrected near visual acuity. In our previous retrospective study, the standard deviations (SDs) of distance-corrected visual acuity after implantation of TNTF00 IOLs were in the range from 0.09 to 0.11 logMAR at 30 and 40 cm [9]. Hence, twenty-three patients were necessary to examine differences of 0.10 logMAR (approximately 1 step in Snellen chart) with a significant level of 0.05 and detection power of 0.85 (package ‘pwr’ version 1.3, R version 3.6.1). Considering a 20% dropout rate in each group, the sample size was calculated to be 30 patients for each IOL group.

### Intraocular lenses and Surgery

The CDF IOLs (models: DFR00V and DFW150/225/300/375) of violet-light-blocked hydrophobic acrylic material had an aspheric optic with a diameter of 6 mm, a continuous sharp edge on the posterior surface, and anteriorly shifted haptics. The diffractive optics were combined with the echelette optics for producing the extended-depth-of-focus function (same as Symfony^®^ IOL, Johnson & Johnson Surgical Vision) and bifocal optics with an add power of 3.50 D for near vision, for producing a continuous depth-of-focus vision. Control IOLs were diffractive trifocal PanOptix^®^ IOLs (TFNT00 and TFNT30/40/50/60) of blue-light blocked hydrophobic acrylic material with an aspheric optic with a diameter of 6 mm and a sharp edge on the posterior surface. The diffractive optics of a diameter of 4.5 mm on the anterior surface produced add powers of 1.25 and 2.5 D. Power of all IOLs was determined for emmetropia with the use of biometry and power calculation formula routinely used at each site.

In surgery, cataract was removed using phacoemulsification and aspiration techniques through a temporal corneal incision of widths of 2.2 to 2.4 mm, and IOL was inserted in the capsular bag using specific injectors.

### Postoperative examinations

Three months after surgery, binocular visual acuity, binocular contrast sensitivity, and binocular defocus curves were examined. Binocular uncorrected and distance-corrected visual acuities (BUCVAs and BDCVAs, respectively) at distances of 0.3, 0.4, 0.5, 0.7, and 5 m were measured using Landolt ring charts under photopic illumination (85–110 cd/m^2^). The manifest refraction spherical equivalent (MRSE) was also measured during the measurement of distance-corrected visual acuity (DCVA) at 5 m. For eyes with continuous depth-of-focus IOLs, DCVA was examined without the use of objective refraction results [10]; increasing the spherical powers in 0.25-D increments until the corrected visual acuity decreased from the best-corrected measurement, and the power before the decrease was recorded. Measured spherical refraction was corrected to infinity by adding − 0.20 D. BDCVAs were measured under correction of MRSE values at 5 m. Visual acuity was converted into logMAR for analysis.

Binocular distance contrast sensitivity was measured using CSV-1000 (Vector Vision, Fairfield, CT) under distance-corrected and photonic illumination (85 cd/m^2^) at a distance of 2.5 m. Logarithm contrast sensitivities at spatial frequencies of 3, 6, 12, and 18 cycle per degree (cpd) were obtained, and the area under the logarithm contrast sensitivity function (AULCSF) was calculated [11] and compared. Photopic binocular contrast sensitivities at 0.4 and 1 m were also measured using the Pelli-Robson charts (Precision-Vison, Woodstock, IL). From the number of characters the patients read, logarithm contrast sensitivities were calculated.

Binocular defocus curves between − 5.00 and + 2.00 D in a step of 0.5 D were measured.

Symptoms of glare, halo, starburst, and waxy vision were assessed using a questionnaire, and the severity was graded on a 5-point scale: not at all, slight: moderate, and very: extreme (impairing daily life). In the questionnaire, satisfaction with near, intermediate, and far vision was assessed similarly.

### Statistical analysis

The primary endpoint of this study was to examine the differences in BDCVA at distances of 0.3 and 0.4 m, photopic symptoms, and satisfaction for each distance vision between eyes with two types of IOLs. Differences in BDCVA for these distances were evaluated using the Mann-Whitney U test. Regarding the photopic symptoms, the proportions of patients with significant symptoms, including moderate-to-severe cases, were compared using the chi-squared test. The rates of satisfaction with near, intermediate, and far vision were analyzed in the same manner. As a secondary endpoint, binocular contrast sensitivity at far, 1 m, and 0.4 m were compared using t-test. Statistical significance was set at P < 0.05.

For presenting the efficacy, the cumulative percentage of patients achieving binocular (20/x or better) at distances of 0.4 m (near), 0.7 m (intermediate), and 5 m (far) for each IOL were plotted [12]. For binocular defocus curves, the areas under the curve (AUCs) below 0.3 logMAR were calculated using a trapezoidal numerical integration method [12, 13], and AUCs for total (T: +2.0 to -5.0 D), far (F: +0.5 to -0.5 D), intermediate (I: -0.5 to -2.0 D), and near (N: -2.0 to -4.0 D) ranges were compared between the two patient groups using t-test.

## Results

**Fig. 1:**
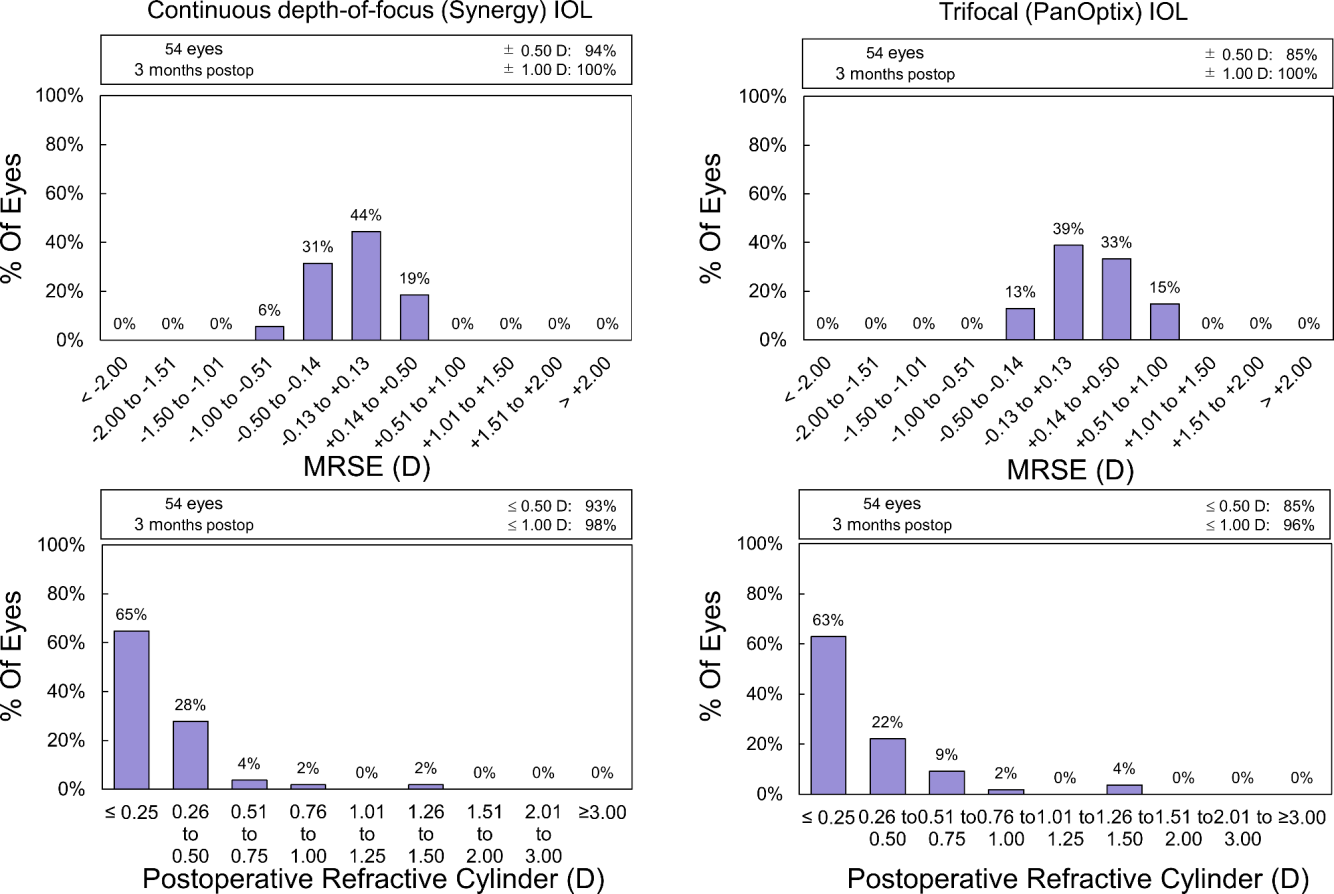
Distribution of MRSE (upper) and refractive cylinder (lower) of eyes with continuous depth-of-focus (Synergy, left) and trifocal (PanOptix, right) IOLs.

Among 60 patients enrolled, four cases were withdrawn, and two cases could not be followed up due to relocation and transfer, so there were 27 patients with CDF IOLs (group S) and 27 patients with trifocal PanOptix^®^ IOLs (group P), eligible for analysis. The demographic data of the eligible patients are listed in Table 1. The mean age and MRSE for infinity were significantly different between the groups, however, the mean differences were 4.6 years and 0.30 D, respectively, which was considered clinically acceptable. Figure 1 shows distributions of the MRSEs and refractive cylinders. While there were 40 eyes with toric models, the mean residual cylinder was − 0.20 (SD:0.27) D, which was comparable with the cylindrical refractions of eyes with non-toric IOLs, -0.26 (SD:0.39 D, P = 0.39, t-test).

**Table 1:** Demographic data of the eligible patients

IOL type	Continuous depth-of-focus (Synergy, group S)	Trifocal (PanOptix, group P)	P value
N	54 eyes of 27 patients	54 eyes of 27 patients	
Mean age, year	66.7 (SD: 4.5) [range: 61–75]	71.3 (SD: 4.9) [range:60–78]	< 0.001*
Man / woman	6 / 21	6 / 21	
IOL model	DFR00V: 33 eyes Toric models DFW150: 14 eyes DFW225: 5 eyes DFW300: 2 eyes	TFNT00: 35 eyes Toric models TFNT30: 16 eyes TFNT40: 2 eyes TFNT50: 1 eye	
Mean MRSE, D	-0.08 (SD: 0.28) [range: -0.75 - +0.50]	+ 0.22 (SD: 0.34) [range: -0.25 to + 1.00]	< 0.001*

*: t-test. IOL, intraocular lens; SD, standard deviation; MRSE, manifest refraction spherical equivalent

**Fig. 2:**
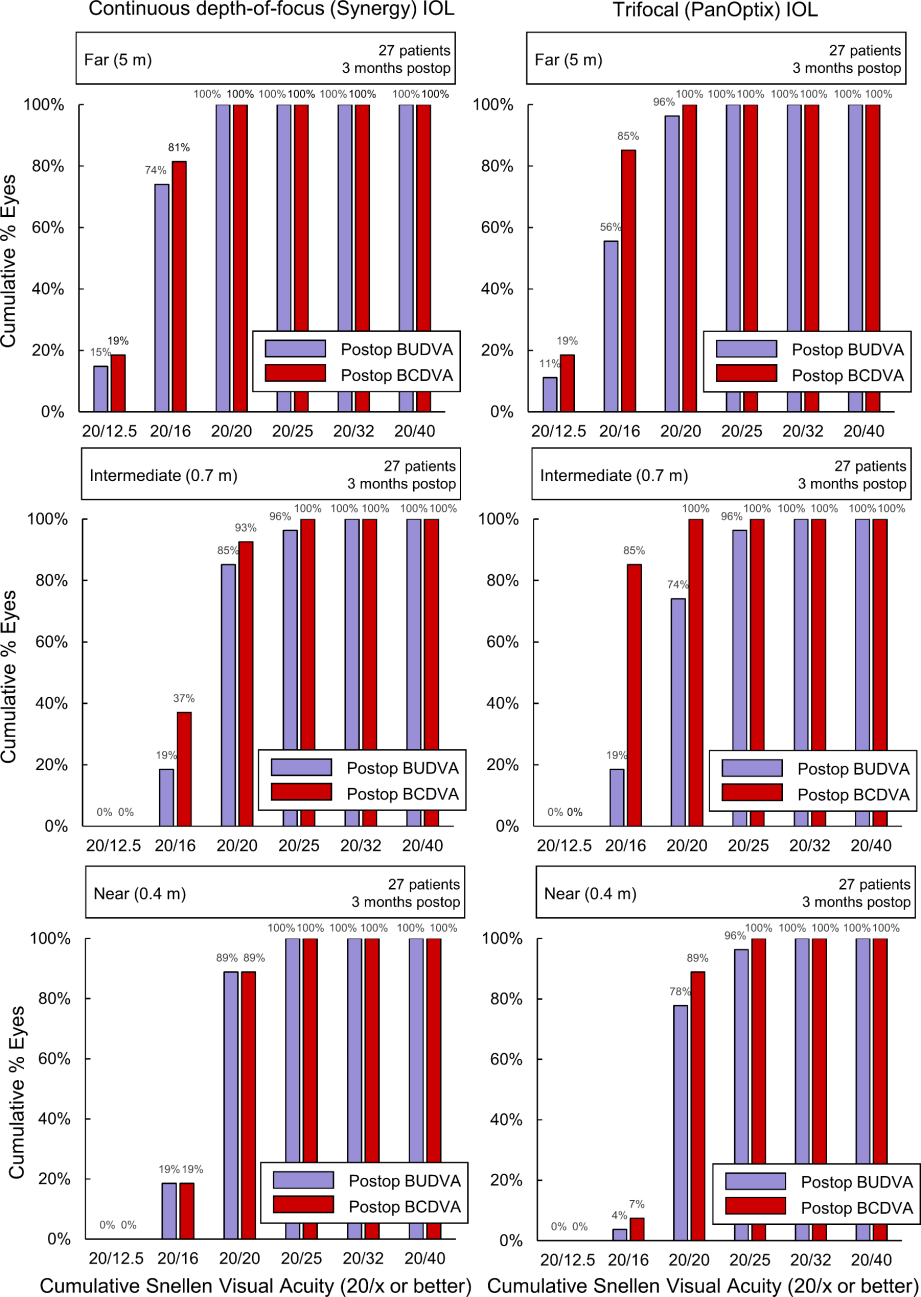
Cumulative percentage of patients achieving BDCVA and BUCVA at distances of 0.4 m (near, bottom), 0.7 m (intermediate, center), and 5 m (far, top) of eyes with continuous depth-of-focus (Synergy, left) and trifocal (PanOptix, right) IOLs.

Table 2 shows the mean BDCVAs and BUCVAs values. While the differences in BDCVA at distances of 0.3 and 0.4 m were one of the primary endpoints, there were no differences (P > 0.20, Man-Whitney U test). Figure 2 shows the cumulative percentage of patients achieving BDCVAs and BUCVAs at distances of 0.4 m (near), 0.7 m (intermediate), and 5 m (far) for each IOL. Significant differences were found in the mean BDCVA at 0.5 m and mean BUCVA at 0.4 m (P = 0.042 and 0.029, respectively, the Man-Whitney U test). The mean differences (0.050 and 0.046 logMAR, respectively) were observed to be at the level of a step of the charts.

**Table 2:** Postoperative mean BDCVAs and BUCVAs at distances of 0.3, 0.4, 0.5, 0.7, and 5 m

	Group S Continuous depth-of-focus IOL	Group P Trifocal IOL	P value*
BDCVA, logMAR			
at 0.3 m at 0.4 m at 0.5 m at 0.7 m at 5 m	+ 0.05 (SD: 0.10) -0.07 (SD: 0.07) -0.10 (SD: 0.08) -0.09 (SD: 0.07) -0.18 (SD: 0.07)	+ 0.04 (SD: 0.08) -0.04 (SD: 0.06) -0.05 (SD: 0.10) -0.06 (SD: 0.08) -0.18 (SD: 0.07)	0.81 0.20 0.042 0.184 0.82
BUCVA, logMAR			
at 0.3 m at 0.4 m at 0.5 m at 0.7 m at 5 m	+ 0.06 (SD: 0.09) -0.07 (SD: 0.07) -0.08 (SD: 0.09) -0.06 (SD: 0.08) -0.15 (SD: 0.09)	+ 0.10 (SD: 0.09) -0.02 (SD: 0.07) -0.04 (SD: 0.10) -0.04 (SD: 0.09) -0.14 (SD: 0.09)	0.11 0.029 0.096 0.29 0.38

*: t-test

BDCVA: binocular distance-corrected visual acuity; BUCVA: binocular uncorrected visual acuity; IOL: intraocular lens; SD: standard deviation

Binocular contrast sensitivity was examined in 26 patients with CDF IOLs and 27 patients with PanOptix® IOLs. Table 3 lists the mean logarithms contrast sensitivities and AULCSF values. When CSV-1000 was used, there were no differences in the mean logarithmic contrast sensitivity at spatial frequencies of 3, 6, 12, and 18 cpd or their AULCSF values (P > 0.092). In the mean logarithm contrast sensitivity at 0.4 and 1 m measured with the Pelli-Robson charts, there were no differences (P > 0.79, t-test).

**Table 3:** Postoperative mean binocular logarithm contrast sensitivities

	Group S Continuous depth-of-focus IOL	Group P Trifocal IOL	P value*
CSV-1000			
at 3 cpd at 6 cpd at 12 cpd at 18 cpd AULCSF	1.75 (SD: 0.18) 1.92 (SD: 0.19) 1.54 (SD: 0.31) 1.08 (SD: 0.29) 1.75 (SD: 0.25)	1.82 (SD: 0.15) 1.93 (SD: 0.17) 1.57 (SD: 0.21) 0.95 (SD: 0.28) 1.85 (SD: 0.18)	0.14 0.70 0.68 0.094 0.092
Pelli-Robson charts			
at 1 m at 0.4 m	1.82 (SD: 0.15) 1.87 (SD: 0.16)	1.81 (SD: 0.12) 1.86 (SD: 0.15)	0.88 0.79

*: t-test. IOL, intraocular lens; cpd, cycles per degree; SD, standard deviation

**Fig. 3:**
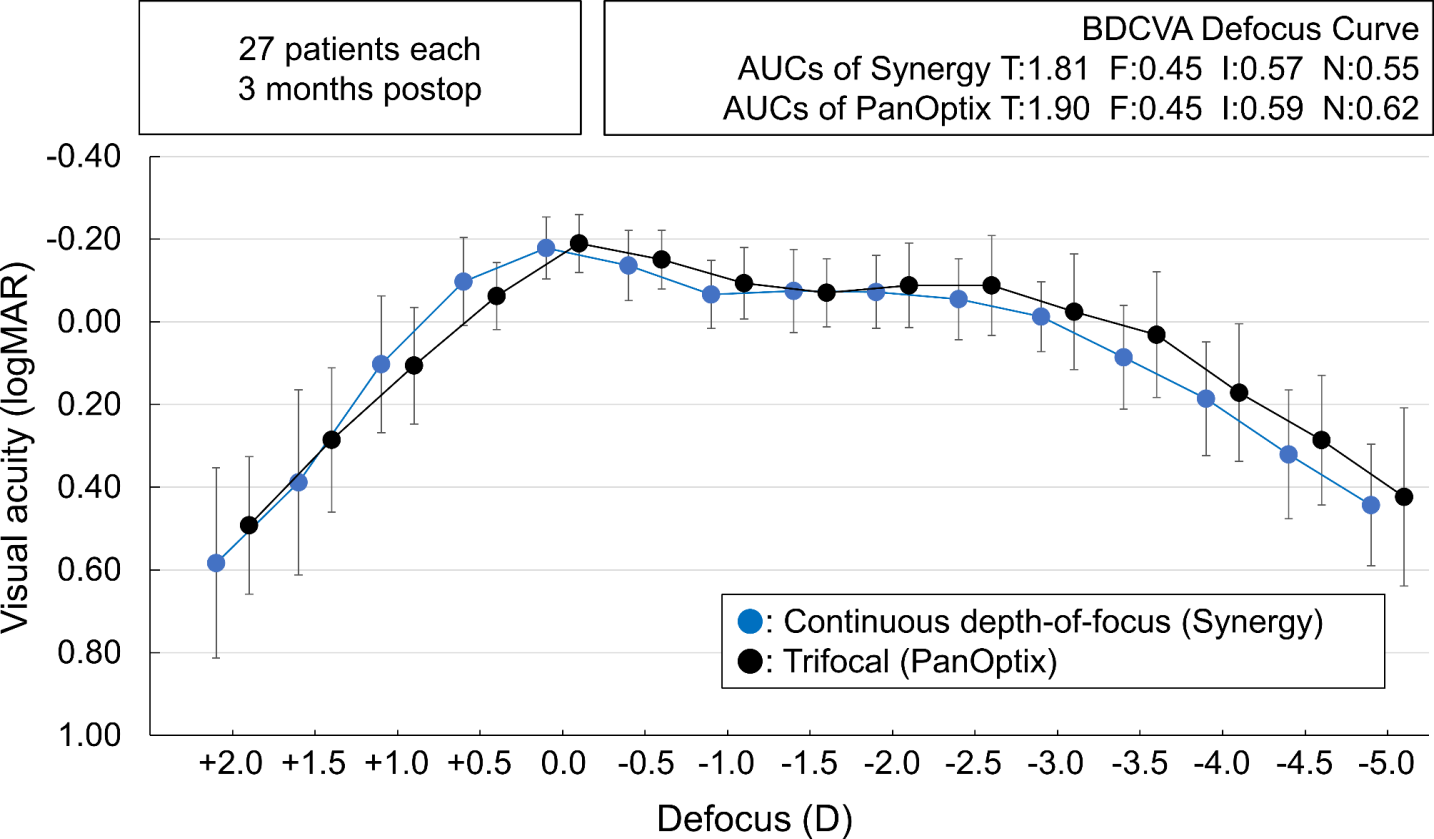
Binocular defocus curve of patients with continuous depth-of-focus (Synergy, left) and trifocal (PanOptix, right) IOLs.

Figure 3 shows the mean binocular defocus curves of patients with the two types of IOLs. There were no differences in the AUCs for the total, far, intermediate, and near ranges (P > 0.31, t-test).

Table 4 lists the number and percentages of patients reporting postoperative symptoms of glare, halo, starburst, and waxy vision. Patients with the CDF IOLs reported more symptoms of very and extreme glare and halos, compared with patients of control IOLs (P = 0.028 and 0.0056, respectively, chi-squared test). Patient satisfaction for far, intermediate, and near visions is shown in Table 5. Higher satisfactions in the near vision were obtained with the use of CDF IOLs (P = 0.0046).

**Table 4:** Number of patients reporting subjective symptoms and their severity

Symptom	Group	Severity				
		Not at all	Slight	Moderate	Very	Extreme
Glare	S	0 (0.0%)	4 (14.8%)	7 (25.9%)	14 (51.9%)	2 (7.4%)
	P	1 (3.7%)	7 (25.9%)	11 (40.7%)	6 (22.2%)	2 (7.4%)
Halo	S	0 (0.0%)	1 (3.7%)	5 (18.5%)	16 (59.3%)	5 (18.5%)
	P	2 (7.4%)	6 (22.2%)	8 (29.6%)	8 (29.6%)	3 (11.1%)
Starburst	S	3 (11.1%)	7 (25.9%)	4 (14.8%)	10 (37.0%)	3 (11.1%)
	P	3 (11.1%)	12 (44.4%)	3 (11.1%)	6 (22.2%)	3 (11.1%)
Waxy vision	S	8 (29.6%)	10 (37.0%)	5 (18.5%)	4 (14.8%)	0 (0.0%)
	P	3 (11.1%)	11 (40.7%)	9 (33.3%)	3 (11.1%)	1 (3.7%)

**Table 5:** Number of patients reporting satisfactions for far, intermediate, and near visions

Distance	Group	Satisfaction				
		Not at all	Slightly	Neutral	Yes	Very
Far	S	2 (7.4%)	1 (3.7%)	1 (3.7%)	13 (48.1%)	10 (37.0%)
	P	0 (0.0%)	1 (3.7%)	3 (11.1%)	10 (37.0%)	13 (48.1%)
Intermediate	S	0 (0.0%)	0 (0.0%)	1 (3.7%)	15 (55.6%)	11 (40.7%)
	P	0 (0.0%)	1 (3.7%)	3 (11.1%)	12 (44.4%)	11 (40.7%)
Near	S	0 (0.0%)	0 (0.0%)	2 (7.4%)	16 (59.3%)	9 (33.3%)
	P	0 (0.0%)	7 (25.9%)	3 (11.1%)	13 (48.1%)	4 (14.8%)

## Discussion

In this prospective comparative study, there were no differences in BDCVA at distances of 0.3 and 0.4 m between groups, while patients in group S experienced more symptoms of glare and halos, and reported higher satisfaction in the near visions. The visual function and optical quality of the same IOLs were evaluated clinically [7] and experimentally [5]. The comparison of BDCVAs at far, intermediate, and near distances by Dick et al. [7], and the current study is shown in Table 6. The results of the 100 eyes with CDF IOLs showed better BDCVAs at far and near distances, whereas such differences were not observed in the current study. An optical bench examination [5] showed that the simulated visual acuity with the use of the two types of IOLs was close under defocus between 0.0 D and − 2.0 D, which coincides with the current results. The mean differences between the groups were 0.045, 0.050, and 0.075 logMAR at far, intermediate, and near distances, respectively. The step of Landolt ring charts used were approximately 0.05 logMAR and BDCVAs were measured under correction of MRSE at 5 m, rather than for infinity. It was speculated that the differences between the previous and current results may be due to differences in measurement conditions and sample size.

**Table 6:** Binocular distance-corrected visual acuities (BDCVAs) of eyes with Synergy and PanOptix IOLs.

Study	Implanted IOL	Far distance	Intermediate distances		Near distances	
		At 4.0 m	At 66 cm		At 40 cm	At 33 cm
Dick et al. [7]	Synergy ZFR00	-0.069 (SD: 0.067)*	+ 0.012 (SD:0.107)		+ 0.025 (SD:0.112)*	+ 0.072 (SD:0.097)*
	PanOptix TNTF00	-0.024 (SD: 0.079)*	+ 0.029 (SD:0.135)		+ 0.075 (SD:0.114)*	+ 0.149 (SD:0.107)*
		At 5.0 m	At 70 cm	At 50 cm	At 40 cm	At 30 cm
Current	Synergy	-0.18 (SD: 0.07)	-0.09 (SD:0.07)	-0.10 (SD:0.08)	-0.07 (SD:0.07)	+ 0.05 (SD:0.10)
	PanOptix	-0.18 (SD: 0.07)	-0.06 (SD:0.08)	-0.05 (SD:0.10)	-0.04 (SD:0.06)	+ 0.04 (SD:0.08)

* Significant difference between the two types of IOLs (P < 0.05)

As for contrast sensitivity, a previous optical bench evaluation showed that the photopic modulation transfer functions (MTFs) at far, intermediate, and near distances were superior when CDF IOLs were used [5]. From this evaluation, better contrast sensitivities at intermediate and near distances were anticipated, however, the current study showed no significant differences between the groups in contrast sensitivity at distances of 0.4 and 1 m in addition to the conventional distance of 2.5 m. Pelli-Robson charts used in the current study are effective for evaluating image contrast but do not examine changes with spatial frequencies. Hence, it was difficult to compare the MTFs as done by optical bench evaluation. Further investigations using sinusoidal grading charts for intermediate and near distances are required.

The symptoms of glare and halos were reported in more patients with CDF IOLs. Previous publications claimed that there was a higher severity of glare [6] and a higher frequency and severity of halo [7]. Light disturbances in the point-spread functions obtained in the optical evaluation indicated relatively stronger halo rings in the CDF IOL [5]. These findings are consistent with our results.

In contrast, satisfaction with near vision was superior in patients with the CDF IOL, though there were no significant differences in the BDCVAs and all-distance contrast sensitivity. Slight postoperative hyperopia of group P might play a role, so that additional evaluation was performed. Table 7 shows the comparison of BUDVA at 30 and 40 cm, contrast sensitivity at 40 cm, and MRSE between the patients who reported not being satisfied for near vision and other patients. With this comparison, it can be assumed that in the current study, hyperopic shifted MRSEs had limited influence on the results. Another possibility would be the difference of Japanese characters and alphabet letters used in the examination. Japanese and Chinese characters require larger font sizes to achieve the same visual acuity for alphabet letters [14], which would be one of the factors. At this time, it was unclear why satisfaction with near vision was higher in patients with the CDF IOLs.

**Table 7:** Comparison of mean binocular uncorrected visual acuities (BUCVAs) at 30 and 40 cm, contrast sensitivity at 40 cm, and manifest refraction spherical equivalent (MRSE) between patients unsatisfied (Not at all) and other patients with PanOptix IOLs.

Patient satisfaction	Unsatisfied (Not at all) (N = 7)	Other (N = 20)	P value
BDCVA at 30 cm, logMAR	0.11 (SD: 0.13)	0.09 (SD: 0.08)	0.71^#^
BDCVA at 40 cm, logMAR	0.00 (SD: 0.07)	-0.03 (SD: 0.07)	0.21^#^
Contrast sensitivity at 40 cm	1.80 (SD: 0.19)	1.88 (SD: 0.12)	0.31*
MRSE, D	+ 0.14 (SD: 0.21)	+ 0.25 (SD: 0.31)	0.45*

^#^: Mann-Whitney test *: t-test; SD, standard deviation

This study had some limitations. First, the mean ages of the two IOLs were significantly different (group S: 66.7 years, group P: 71.3 years). When looking at the influence of patient age on postoperative visual acuity after multifocal IOL implantation, there were no significant differences in visual function between the 60 and 70 s [15]. Although this study by Yoshino et al. supports our findings that the influence would be minimum, this can be further investigated with a larger sample size. Second, the significant difference in MRSE was found. The spherical and cylindrical powers were measured in increments of 0.25 D and the mean difference of 0.30 D was considered within the measurement tolerance. However, it would be ideal to compare with MRSE that has no significance. Third, diffractive IOLs using echelette gratings, such as Synergy^®^ IOL, induce constant differences in subjective and objective refractions [10]. Thus, the results of objective refraction should not be used as a reference in examining the DCVA to avoid incorrect MRSE. Lastly, sinusoidal grading charts were not available for comparison of CS. Such a chart is effective for comparing the MTF at each distance and investigating the image quality of intermediate and near vision.

## Conclusions

The binocular visual functions of patients with continuous depth-of-focus IOLs were comparable to or better than those of patients who received trifocal IOLs. Although the glare and halo were enhanced, the patient was satisfied with near vision. Understanding the differences between the two types of presbyopia-correcting IOLs is important to ensure patient satisfaction.

## Data Availability

The datasets used and/or analyzed in the current study are available from the corresponding author upon reasonable request.

## List of abbreviations

AUC: areas under the curve
AULCSF: area under the logarithm contrast sensitivity function
BDCVA: Binocular distance-corrected visual acuity
BUCVA: Binocular uncorrected visual acuity
CDF: continuous depth-of-focus
CRB: Certified Review Board
cpd: cycle per degree
DCVA: distance-corrected visual acuity
IOL: intraocular lens
logMAR: logarithm of the minimum angle of resolution
MRSE: Manifest refraction spherical equivalent
MTF: modulation transfer function
SD: standard deviation
